# Risk Factor and Cluster Analysis to Identify Malaria Hot Spot for Control Strategy in Samigaluh Sub-District, Kulon Progo, Indonesia

**Published:** 2019-09

**Authors:** Sulistyawati SULISTYAWATI, Isnah FITRIANI

**Affiliations:** Department of Public Health, Universitas Ahmad Dahlan, Yogyakarta, Indonesia

## Introduction

Risk factor assessment and hot-spot analysis is required reckoning prior determining malaria control strategy due to the program effectiveness and cost efficiency reason. All the time, a lot of resources including human and money have to pay to prevent malaria transmission worldwide. Malaria which is caused by *Plasmodium* and transferred by *Anopheles* mosquitos has become major public health problem worldwide. Even though WHO said that globally the number of malaria incidence was decreased regarding the strong effort in malaria prevention. In fact, most a half of world population is still at risk of malaria and estimated there were 212 million malaria cases in 2015 with 429.000 mortality (1).

**Fig. 1:**
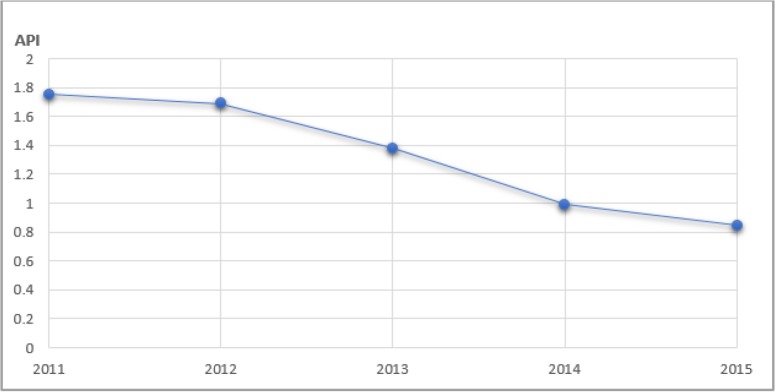
Trend of API malaria in Indonesia between 2011–2015 (1)

In Indonesia, Annual Parasite Incidence (API) of malaria tends to decrease during 2011 until 2015 as shown in Fig. 1. But, some regions in the east of Indonesia such as Papua, West Papua, West Nusa Tenggara, Maluku, North Maluku, Bengkulu, Bangka Belitung and North Sulawesi is still suffering from Malaria, thus was indicated by their API which is above the national level (0.85) (2).

In the west part of Indonesia, Java Island is one of malaria hypoendemic (3), including Kulonprogo District where Samigaluh is present as one of sub-district with Low Case Incidence (LCI) in 2011 (4).National policy has set up their target to eliminate malaria in Java by 2015 by implementing several malaria national programs (5). Nevertheless, malaria case still found until 2017, including in Samigaluh II, Kulonprogo. Personal communication with malaria programmer in Kulonprogo district health office revealed that by 2017 Samigaluh II owning of High Case Incidence (HCI) of malaria due to the increase of new malaria case. Thus, illustrated that malaria in Java including Samigaluh, Kulonprogo requires more attention.

Cluster analysis was a highly powerful in malaria control across the globe (6–8). While in Indonesia, insufficiently research discuss the using of cluster analysis in malaria control. May this be a reason that cluster analysis method did not take into account by the health authorities on their malaria control strategy.

Contemplation to the whole background, this research was aimed to assess malaria risk factor in Samigaluh II PHC and performed cluster analysis as supporting tools. This research is a crucial step in malaria control to show, communicate and direct the local authorities to adopt this method on their decision-making.

## Materials and Methods

This research was a quantitative analytical observational by case-control approach supported by Geographic Information System. Population for this research was 208 people who took blood examination in Samigaluh II Public Health Center (PHC) II, Kulon Progo, Indonesia. Total sample was applied to select the cases. Case was defined as everyone who diagnosed as positive malaria through blood examination in Samigaluh II PHC during January–December 2016. Control was everyone who diagnosed as negative malaria through blood examination in Samigaluh II PHC during January–December 2016”.

**Fig. 2:**
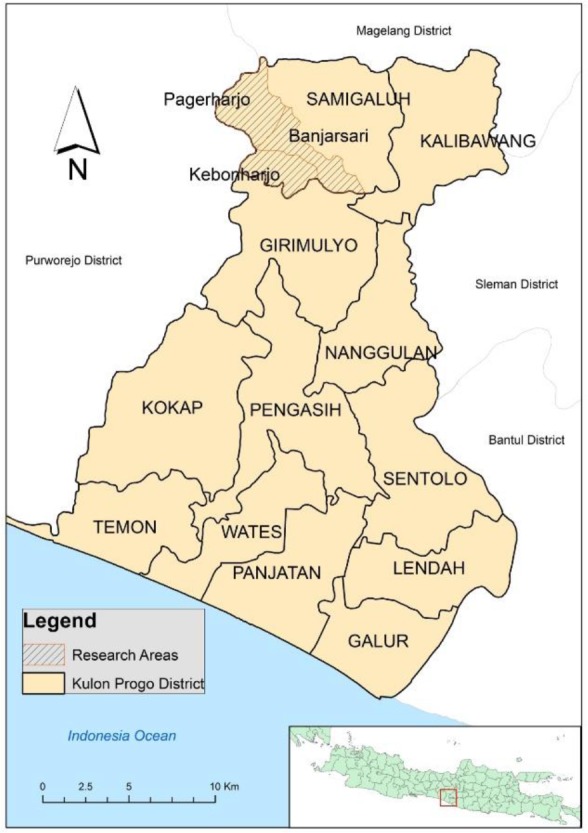
Research Areas, which were consisted of 3 villages: Pagerharjo, Banjarsari and Kebonharjo, Samigaluh, Kulon Progo, Yogyakarta Province, Indonesia

This research was conducted during January–July 2017 in Samigaluh II PHC, Kulon Progo, Yogyakarta, Indonesia (Fig. 2). Prior the research, information about the research was given to the participants including their freedom to quit from this research anytime without any penalty. For people who agreed to participate in this research, a written informed consent was requested from them.

A total 86 participants were joined in this research, which was comprised of 43 cases and 43 controls. Informed consent was collected before the study started. This research received approval from the Scientific Board Committee of the Public Health Department UAD.

A structured questionnaire, checklist, and Geographic Positioning System (GPS) were used to collect the data. A questionnaire was established by the researcher based on literature review then tested on 30 people who were not included in our sample with 0.675 Cronbach’s Alpha. Questionnaire divided into two sections: First was general questions (Name, Sex, Age, Address, and Occupation). A second part was multiple choice questions related to malaria prevention such as habit in using a bed net, repellent, outdoor activity and other risk factors. Checklist was used as guidance when observed the existence of ventilation net, livestock cage, and for writing the coordinate the case and control. For every variable, participants divided into two categories (at risk and not risk) based on their answer.

An analysis was conducted two analyses, which were statistical analysis and cluster analysis. Descriptive, Bivariate and Multivariate analyses were performed by SPSS version 1.6. Probability was counted based on p = 1/ (1 + e^−y^) formula, where p is probability, e is a natural number, and y is constant. Cluster analysis was generated by The Kulldorff spatial scan statistic, using SaTScan TM version 9.4 software (http://satscan.org) to test the spatial clustering. A purely spatial analysis was used in this research based on the Bernoulli probability model that is appropriate for 0/1 event data such as cases/controls.

## Results

A total 86 participants enrolled in this study, all of which majority (54.65%) was male. Most of the participants (18.60%) aged 36–45 yr old. Among the participants, the majority (33.72%) graduated from senior high school. Summary participants’ characteristic is presented in Table 1.

**Table 1:** Participant characteristics by sex, age and education

***Characteristics***	***n***	***Percentage (%)***
Sex		
Male	47	54.65
Female	38	45.35
Age group (yr)				
0–5	2	2.33
5–11	10	11.63
12–16	5	5.81
17–25	11	12.79
26–35	14	16.28
36–45	16	18.60
46–55	13	15.12
56–65	6	6.98
>65	9	10.46
Education		
No school	2	2.33
Pre-school	5	5.81
Primary school	22	25.58
Junior high school	25	29.07
Senior high school	29	33.72
University education	3	3.49

Source: Primary data

Malaria risk factor was explained by six variables: occupation, ventilation net, bed net usage, the presence of livestock cage, repellent usage and presence of night outdoor activity. Among variables, occupation was the only variable that correlating with malaria case (*P*= 0.031). Taking into account the Odd Ratio (OR), five variables which were the presence of ventilation net, occupation, the presence of bed net, repellent usage and habit of doing outdoor activity in the night, were detected as a risk factor. The presence of livestock cage was a protective factor. Considering *P*-value = 0.25, two variables which were an occupation and having nigh outdoor activity were included in logistic regression analysis (Table 2).

**Table 2:** Bivariate analysis malaria Risk factor and malaria Incidence

***Variable***	***Cases (n= 43)***	***Control (n=43)***	***P value***	***Odds Ratio***	***Confidence Interval (95%)***
Occupation					
At Risk	26	15	0.031	2.855	1.189–6.854
No Risk	17	28			
Ventilation Net			1.000	1.232	0.346–4.389
At Risk	38	37			
No Risk	5	6			
Bed Net			0.635	1.404	0.551–3.551
At Risk	32	29			
No Risk	11	14			
Presence of livestock cage ≤ 50 meters from the house					
At Risk	9	13	0.550	0.611	0.229–1.630
No Risk	34	30			
Repellent usage			0.626	1.430	0.547–3.740
At Risk	33	30			
No Risk	10	13			
Doing outdoor activity in the night			0.144	2.955	08.48–10.300
Yes	10	4			
No	33	39			

Source: Primary data

**Fig. 3:**
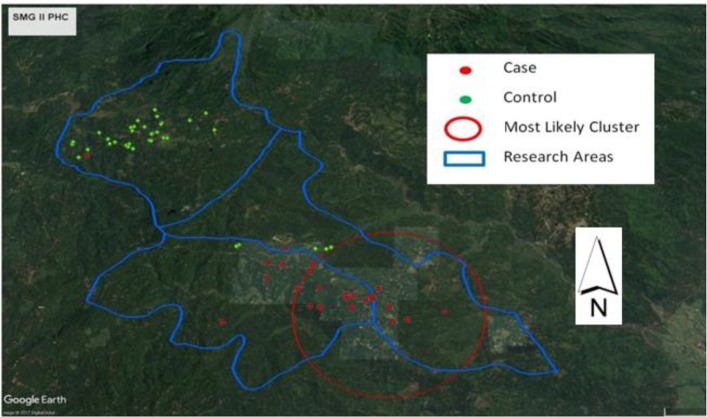
Overlay of Google Earth Imagery with cluster and participant position

Figure 3 shows malaria cluster during research period in Samigaluh II PHC, Kulonprogo. Satscan detected one significant cluster with a p-value less than 0.05 and radius 1.51 km on the research area. This cluster located in Kebonharjo and Banjarsari villages. Table 3 presents stepwise logistic regression done to see the most associated variable to malaria. In the first step occupation and doing outdoor activity were included in the analysis. Subsequently, considering the *P*-value, the only occupation went to second step analysis. Based on probability p = 1/ (1 + e^−y^) formula, the probability to get malaria infection for those at occupation at risk as follows:

$$\begin{array}{l} \text{y}=-0,499+1,049\times \text{occupations}\;\text{at}\;\text{risk}\\ \text{y}=0,55\\ \text{p}=1/(1+{\text{e}}^{-\text{y}})=|1/(1+2,7^{-0,55})=0,63=63\%. \end{array}$$

Meaning that people who have an occupation as a lumberman, farmer, breeder, sand miner, sugar palm tapper, and carpenter owned 63% probability to get malaria infection than others occupation such as civil servant, tradesman, and student.

**Table 3:** Logistic Regression Analysis

***Variable***	***B***	***P value***	***Exp. (B)***	***Confidence Interval (95%)***	
				***Lower***	***Upper***
Step 1					
Occupation	0.913	0,048	2,492	1,009	6,154
Doing outdoor activity in the night	0.779	0,241	2,179	0,593	8,005
Step 2					
Occupation	1.049	0,019	2,855	1,189	6,854
Constanta	−0.499	0,105	0,607		

## Discussion

Malaria is emerging disease and has become public health problem particularly in tropical countries such as Indonesia. Although many prevention programs were launched by government and eradication target has been made, but in fact, the number of malaria keep steady in this country. Samigaluh sub-district, Kulon Progo is one of endemic malaria area in Indonesia (9), particularly in Java Island. This district becomes endemic due to the position in adjacent with other malaria endemic area, such as Purworejo and Magelang (4, 10). Kulon Progo health authority has done routine prevention effort also has collaboration with a researcher to formulate appropriate malaria prevention, but until 2016, malaria cases sustain on this area. Accordingly, a holistic action is needed. Plenty of research has been done regarding malaria risk factor assessment both in Indonesia and Kulonprogo (11, 12), but few of them that combining with cluster analysis. Therefore, this research tried to explore risk factor of malaria in the research area with statistic and cluster analysis method to facilitate in developing proper malaria intervention for the health authorities.

In this research, we measured the malaria risk factor using statistic and strengthened by cluster analysis to provide factual evidence in the research area. Through questionnaire and geo-position plotting, we identified that outdoor occupation was the only risk factor that associated to malaria incidence. Afterward, Satscan analysis recognized one cluster as the center of malaria transmission in Samigaluh II PHC. Outdoor occupation is increasing people to have a risk of malaria (13, 14), including for people who have outdoor activity (15). This finding was consistent with this research outcome. Many people in Samigaluh II work as a lumberman, farmer, breeder, sand miner, sugar palm tapper and carpenter, which was usually performed their work up to late night.

Based on information from District Health Officer and strengthened, the main vector on Samigaluh was *An. balabacensis* and *An. maculatus* (9). Those mosquitos have biting activity all night and belong to some peak during that time (16). Accordingly, people who have activity in the night predicts will increase their risk to infected by Anopheles biting. This finding was approved by research in Ghana which reported that there was a relationship between night outdoor activities and malaria (17).

Cluster analysis was used in several malaria research (3, 18, 19), some of them prove that cluster analysis was potentially tool to guide determining malaria intervention strategy by the policymaker. Refer to Satscan analysis; there was a significant cluster in the southeast of Samigaluh II that fall in the malaria hot spot (Fig. 2). Samigaluh is hilly sub-district (4); people live in the settlement which is majority presence of multiple agriculture on their field (20). This statement was confirmed by the screenshot from Google Earth that shown majority of the area is in green colour indicating the plantation. Additionally, close to the cluster, presence the river that possibly as breeding place of the mosquitos. Accordingly, several factors may cause the cluster significant: first was the existence of proper *Anopheles* habitat and second was the ownership of risky occupation to malaria infection. In short, translating cluster finding on the implementation section was done by considering the number of the household included as in Satscan result. This number reflected the malaria prevalence on this cluster (13).

It is important to understand how to interpret this result in different areas. Human and geography condition always change along the time, so that the influencing to the analysis is may occur due to these dynamic processes. Our research which included human socio-cultural and cluster analysis as a representation of environment factor was conducted in a small setting and did not provide plotting the breeding site as geo analysis. Also, the cluster may be different if analysis conducted in aggregated data and different spatial scale. Considering this limitation, we propose in the future research to discuss the benefit of using cluster analysis in malaria control strategy and the real implementation should be tested. However, this research emphasizes the finding on the occupation as the only risk factor to malaria in the research area. Education and promotion of the population at risk should be made to increase their awareness.

## Conclusion

Malaria is emerging disease, although many control efforts have been made in fact, the case still occurs. Accordingly, it is important to have an additional approach to determine priority area as malaria control target. In this case, we employed Satscan analysis as our additional information besides the statistical analysis. At the end of the research, we found that occupation was the only risk factor for malaria infection. Moreover, established a significant cluster that falls on the malaria hotspot. By considering the science development, it is important to have integrated approach to solving the problem, including in malaria problem.

## Ethical considerations

Ethical issues (Including plagiarism, informed consent, misconduct, data fabrication and/or falsification, double publication and/or submission, redundancy, etc.) have been completely observed by the authors.
